# Excess serum uric acid is associated with metabolic syndrome in obese adolescent patients

**DOI:** 10.1007/s40200-020-00507-2

**Published:** 2020-05-16

**Authors:** Christy Foster, Loretta Smith, Ramin Alemzadeh

**Affiliations:** 1 Division of Endocrinology, Department of Pediatrics, University of Alabama at Birmingham, 1601 4th Avenue South, Birmingham, AL USA; 2 Division of Endocrinology, Department of Pediatrics, University of Tennessee Health Science Center, Memphis, TN USA

**Keywords:** Hyperuricemia, Inflammation, Adolescents, Metabolic syndrome, Obesity

## Abstract

**Purpose:**

Obesity is a significant cause of morbidity in adolescents. Excess serum uric acid (SUA) has been associated with metabolic syndrome (MS) among adults. We evaluated the relationship among SUA and markers of insulin resistance (IR) and low-grade inflammation in obese adolescents with and without MS.

**Methods:**

The study was a retrospective chart review of obese patients seen in the LeBonheur Endocrine clinic seen in clinic between September 2016 and December 2017. MS was defined as according to the International Diabetes Federation. Body mass index standard deviation score (BMI SDS), systolic blood pressure (SBP), diastolic blood pressure (DBP), body composition, fasting lipids, glucose, high sensitivity c-reactive protein (hs-CRP), serum uric acid (SUA), HbA1c, alanine transferase (ALT), aspartate transferase (AST), insulin and homeostatic model assessment for insulin resistance (HOMA-IR) were extracted from the charts of the 100 obese adolescents (57% female).

**Results:**

Hyperuricemia (SUA >357 umol/L) was present in 41.8% of entire cohort without significant ethnic/racial and/or gender differences. Adolescents with HUA had higher FM, SBP, HbA1c, insulin and HOMA-IR (*p* < 0.05). While SUA was positively correlated with FM, SBP, HOMA-IR and HbA1c, and triglyceride:HDL-C ratio (TG:HDL-C) (p < 0.05). MS was identified in 32.8% of cohort. MS showed significantly higher FM, SBP, DBP, SUA, ALT, insulin, HOMA-IR, and TG:HDL-c ratio than non-MS subgroup (*p* < 0.05). FM was positively correlated with SUA, HOMA-IR and hsCRP (*p* < 0.01).

**Conclusions:**

In our study, those with hyperuricemia (HUA) showed elevated markers of metabolic syndrome including BP, serum glucoses, IR and triglycerides. In our cohort, SUA appears to correlate with MS comorbidities.

## Introduction

Obesity has been identified as significant public health concern over the past couple decades [1]. Metabolic syndrome (MS) is a cluster of symptoms including central obesity, insulin resistance, hypertension, and hypercholesterolemia [2]. According to the World Health Organization, the worldwide prevalence of obesity in children has increased by more than three fold over the past four decades [3]. Metabolic syndrome in children is a risk factor for development of Type 2 diabetes and adult MS [4], therefore more studies are needed to understand the pathophysiology of MS leading to inflammation. In addition, serum uric acid (SUA) has been found to be an independent risk factor for MS [5]. However, SUA homeostasis and its relationship with MS are considered complex [6]. High levels of SUA will lead to sequelae including hypertension [7]–[8], hypertriglyceridemia, and hypercholesterolemia [5, 8].

Uric acid is the end product of purine metabolism and is secreted by the kidney. Elevated levels of SUA can result from decreased renal clearance. Hyperinsulinemia has been postulated to decrease uric acid clearance by the kidneys and to increase serum uric acid [9]. Nitric oxide (NO) is the major endothelium-derived relaxing factor associated with oxidative stress and insulin resistance. Uric acid impairs endothelial function and enhances nitric synthase deficiency, which reduces NO, a known mechanism for inducing insulin resistance [10]. Also, SUA has been found to be associated with cardiovascular disease in adults with or without impaired glucose tolerance [11].

Hyperuricemia has been shown to be associated with MS in adult patients. Adults with increasing BMI over time show increased SUA and high sensitivity C-reactive protein (hsCRP) [12]. Serum uric acid has also been shown to correlate with liver dysfunction and increased inflammatory markers [10, 13–17] and negatively correlate with fasting blood glucose, serum HbA1c and HDL-C [18]. Prior studies define hyperuricemia at serum uric acid level as greater than 6.0 mg/dL. [7]

In obese children, SUA, hsCRP and other inflammatory markers are elevated [19], which has been associated with excess weight and low cardiovascular fitness [20]. Prior studies have looked at SUA level in adolescent patients with metabolic syndrome and have shown a correlation between SUA and markers of inflammation and endothelial dysfunction in pre-pubertal children [19, 21]. It has been shown that markers of inflammation and SUA are associated with biomarkers of insulin resistance and sequela of MS in adolescents [21]. African American adults have significantly higher levels of SUA [22], whereas studies evaluating this in African American adolescents are scarce. Indeed, the metabolic interaction between SUA and low grade inflammation among adolescents has not been fully explored [23].Therefore, we examined the differences in SUA and hsCRP and indices of insulin resistance among adolescents with and without metabolic syndrome.

## Subjects and methods

### Subjects and design

The study was designed as a retrospective chart review of patients seen in the LeBonheur Endocrine clinic from September 2016 to December 2018 for obesity, identified by a practitioner seeing the patients in the LeBonheur clinic who had a weekly obesity clinic. Because this was a retrospective chart review, a sample size calculation was not done. One hundred adolescents (age 13.9–18.9 years old) who met the criteria for obesity [body mass index (BMI) >95th percentile for age] were identified during this period of time. Race was self-assigned: Caucasian (*n* = 28) and African-American (*n* = 72). Children were excluded if they had a diagnosis of diabetes given that this could be a confounding variable, hepatic or renal disease (renal disease defined as GFR <60 ml/min/1.73 m2) metabolic rickets, malabsorptive disorders (Crohn’s disease, cystic fibrosis, and celiac disease) or cancer or were taking anticonvulsants, or systemic glucocorticoids. The UTHSC Institutional Review Board committee approved the retrospective review of patients’ clinical charts; therefore, informed consent was not required.

As part of routine care, participants and/or their guardians completed an intake form detailing their medical history and medications. A well-trained clinician determined pubertal maturation (Tanner stage). Data were collected on patients including age, gender, self-declared race, and anthropometric measurements such as BMI, height, weight, blood pressure, and body composition analysis. Waist circumferences (WC) were measured to the nearest 0.1 cm at the level of iliac crest while the patient was at minimal respiration. Body composition analysis was done using a Tanita DC 430 U body composition impedance scale (Arlington Heights, IL) to measure fat mass (FM), fat free mass (FFM) and total body water (TBW) [24, 25]. Fasting samples for glucose, SUA, insulin, hemoglobin A1C, lipid profile, ALT, AST and hsCRP were obtained between 7 AM-11:30 AM. depending on the patient appointment time.

#### Laboratory studies and calculations

All blood samples were obtained between 7 AM and 11:30 AM after an overnight fast. Serum glucose. Was measured using an autoanalyzer (Ortho-Clinical Diagnostic, Inc. Rochester, NY). Fasting serum insulin was measured using the Architect insulin assay which is a chemiluminescent micro particle immunoassay. Intra-assay and inter-assay CV are 1.7–4.0% and 1.9–4.6% respectively. The homeostatic model assessment of insulin resistance (HOMA-IR) [(blood glucose,mM X insulin,uU/mL)/22.5] was calculated [26]. Hemoglobin A1c was determined using a Vitros Chemistry instrument (non-diabetic range 4%–6%).

Total cholesterol, high-density lipoprotein cholesterol (HDL-C), low-density lipoprotein cholesterol (LDL-C) and triglycerides were determined by colorimetric methods (Vitros 5600 Integrated System; Ortho-Clinical Diagnostics, Inc. Rochester, NY). Low density lipoprotein cholesterol was calculated using Friedewald’s equation [27]. AST and ALT were determined by colorimetric methods (Vitros 5600 Integrated System; Ortho-Clinical Diagnostics, Inc. Rochester, NY).

SUA levels were measured by oxidation with enzyme uricase to form allantoin and hydrogen peroxide by a colorimetric assay (Vitros 5600 Integrated system; Ortho-Clinical Diagnostics, Inc. Rochester, NY). Hyperuricemia was defined as levels above the measurement of 357 uM (6.0 mg/dL) for adolescents aged 13–18 years old as shown in a prior study [7].

Serum hsCRP level was determined by an immunoassay using a polystyrene particle-enhanced immunonephelometric method (Vitros 5600 Integrated system; Ortho-Clinical Diagnostics, Inc. Rochester, NY) The limit of detection was 0.1 mg/L with a measuring range of 0.2–15.0 mg/L. Inter-assay CV was 1.2–5.0%. The hs-CRP values of >10 mg/L were excluded to avoid influence of acute infection [28].

Blood pressure measurements were taken twice with the patient in the sitting position. Elevated systolic blood pressure (SBP) or diastolic blood pressure (DBP) were defined as a value above the 95th percentile for age, gender and height percentile.

MS was defined according to a consensus statement from the International Diabetes Federation. The IDF defines MS as central obesity (defined as a waist circumference > 95th percentile) and two of the following: Triglycerides >150 mg/dL, HDL-C < 40 mg/dL in males or < 50 mg/dL in females, elevated blood pressure (BP >95th percentile based on height, age and gender) and fasting plasma glucose >100 mg/dL [29]]. Adjustments were made regarding the definition of hypertension given that these were children and adolescents in the study. Impaired fasting glucose was defined as glucose between 5.6 mmol/L to 6.9 mmol/L.

### Statistical analyses

Statistical analyses were carried out using SPSS 25. Data are expressed as mean ± standard deviation. BMI values were converted into standard deviations scores (SDS) that were normalized for age and gender based on 2000 Centers for Disease Control (CDC) growth charts. The natural logarithmic transformation of the variables was used in the correlation and regression analyses when the data was found to be skewed. Differences between normal uric acid (NUA) and high uric acid (HUA) groups and those with MS and non-MS groups were estimated using unpaired student t-tests and the *p* value for 2-tailed significance given the direction of the change between the groups was unknown. Chi-squared analyses were used to compare prevalence of hypertension and metabolic syndrome. Spearman correlations were performed to examine associations between SUA, hsCRP, FM, blood pressure, HOMA-IR and triglycerides:cholesterol ratio and triglycerides:HDL ratio. *P* < 0.05 was considered significant.

## Results

### Findings stratified according to presence or absence of elevated serum uric acid

Hyperuricemia was identified in 39% of the cohort. Those patients with HUA also were found to have significantly higher SBP and DBP, BMI SDS, WC, and FM. Total Body Water (TBW) and Free Fat Mass (FFM). Patients with HUA had significantly higher fasting glucose, fasting insulin, HOMA-IR, GFR, and total cholesterol and LDL-C levels compared to those with NUA. Serum aspartate aminotransferase (AST) levels were also significantly higher in those with high SUA (Table 1).

**Table 1:** Clinical and biochemical characteristics of participants based on uric acid status

Parameters	All	HUA (≥357 umol/L)	NUA (<357 umol/L)	*P*
N (%)	100	39	61	NA
Age (yrs)	13.9 ± 2.0	14.4 ± 2.1	13.6 ± 2.0	0.049
Gender (% Female)	57%	46%	64%	NS
Tanner Stage	4.0 ± 1.3	4.4 ± 1.0	3.8 ± 1.4	0.031
Race/Ethnicity				
Caucasians (%)	28%	23%	31%	NS
African Americans(%)	72%	77%	69%	NS
SBP (mmHg)	116.8 ± 10.8	122.2 ± 11.3	113.4 ± 9.0	<0.001
DBP (mmHg)	71.9 ± 8.1	73.9 ± 9.9	70.6 ± 6.4	<0.001
Hypertension %	17%	28%	10%	0.017
BMI SDS	2.4 ± 0.4	2.5 ± 0.3	2.3 ± 0.4	<0.001
Waist Circumference (cm)	108.1 ± 15.4	113.4 ± 13.5	104.7 ± 15.6	0.004
Fat Mass (FM)	42.8 ± 17.6	48.8 ± 17.7	39.0 ± 16.4	0.008
Glucose (mmol/L)	4.8 ± 0.5	4.9 ± 0.5	4.7 ± 0.4	0.038
Insulin (pmol/L)	194.4 ± 157.2	244.3 ± 156.0	162.5 ± 150.8	0.011
HOMA-IR (mol uU/mL)	5.81 ± 4.85	7.50 ± 5.19	4.72 ± 4.32	0.007
GFR (ml/min/1.73 m2)	107.7 ± 19.37	101.7 ± 18.50	111.60 ± 19.08	0.013
AST	29.7 ± 10.5	33.1 ± 12.6	27.5 ± 8.32	0.017
ALT	35.8 ± 16.1	38.05 ± 16.0	34.4 ± 16.2	NS
hs-CRP (mg/L)	2.8 ± 2.2	3.0 ± 2.3	2.6 ± 2.1	NS
SUA (umol/L)	341.5 ± 76.3	414.1 ± 51.8	295.2 ± 47.9	<0.001
Cholesterol (mmol/L)	4.0 ± 0.8	4.2 ± 1.0	3.9 ± 0.7	NS
Triglycerides (mmol/L)	1.2 ± 0.6	1.3 ± 0.6	1.1 ± 0.6	NS
Triglycerides: HDL-C Ratio	2.5 ± 1.5	2.6 ± 1.3	2.4 ± 1.6	NS
Prevalence of MS (%)	30%	46%	20%	0.005

Abbreviations: Not applicable (NA), not significant (NS), serum uric acid (SUA), normal uric acid (NUA), high uric acid (HUA)

Looking at those who were in the lowest and highest quartile of SUA, there was significant difference between BMI SDS, FM, FFM, SBP, and cholesterol/HDL ratio. In the highest quartile of SUA, hsCRP correlated positively with serum glucose. SUA did not correlate with FM, HOMA-IR or SBP (data not shown).

### Findings stratified according to presence or absence of metabolic syndrome

In our cohort, 30% of adolescents met diagnostic criteria for MS. Patients with MS were shown to have significantly higher SBP, DBP, BMI SDS, WC, FM, fasting insulin level, SUA, ALT, total cholesterol, LDL-C, and HOMA-IR. (Table 2).There was no significant difference in GFR between those with metabolic syndrome and those without metabolic syndrome.

**Table 2:** Clinical and Biochemical Characteristics of Participants with and without MS

Parameters	All	MS	Non-MS	***P***
N	100	30	70	
Age (yrs)	13.9 ± 2.0	14.0 ± 2.1	13.9 ± 2.0	NS
Gender (% Female)	57%	67%	53%	NS
Tanner Stage	4.0 ± 1.3	4.2 ± 1.1	3.9 ± 1.3	NS
Race/Ethnicity				
Caucasians (%)	28%	36%	23%	NS
African Americans (%)	72%	63.3%	75.7%	NS
SBP (mmHg)	116.81 ± 10.84	123.6 ± 13.79	113.9 ± 7.75	0.001
DBP (mmHg)	71.9 ± 8.1	75.6 ± 9.8	70.3 ± 6.7	0.011
% Hypertension	17%	37%	9%	0.001
BMI SDS	2.4 ± 0.4	2.5 ± 0.3	2.3 ± 0.4	0.013
Waist circumference	108.1 ± 15.4	112.7 ± 12.8	106.2 ± 16.0	0.033
Fat Mass (kg)	42.8 ± 17.6	48.48 ± 16.2	40.3 ± 17.6	0.028
Glucose	4.8 ± 0.5	4.9 ± 0.6	4.7 ± 0.4	NS
Insulin (pmol/L)	194.4 ± 157.2	296.1 ± 218.4	150.8 ± 94.0	0.001
HOMA-IR	5.8 ± 4.9	8.7 ± 6.7	4.5 ± 2.9	0.001
AST	29.7 ± 10.5	31.9 ± 10.7	28.8 ± 10.4	NS
ALT	35.8 ± 16.1	43.8 ± 22.2	32.4 ± 11.3	0.011
hs-CRP (mg/L)	2.7 ± 2.2	3.1 ± 2.1	2.6 ± 2.2	NS
SUA (umol/L)	341.5 ± 76.3	370.8 ± 77.4	329.0 ± 72.8	0.009
Cholesterol (mmol/L)	4.0 ± 0.8	4.5 ± 0.9	3.8 ± 0.8	<0.001
Triglycerides (mmol/L)	1.2 ± 0.6	1.64 ± 0.7	0.98 ± 0.4	<0.001
Triglycerides: HDL-C Ratio	2.5 ± 1.5	3.7 ± 0.9	1.9 ± 0.9	<0.001

Abbreviations: fat mass (FM), homeostatic model assessment estimates for insulin resistance (HOMA-IR), metabolic syndrome (MS), not applicable (NA) and not significant (NS)

### Findings stratified according to ethnicity/race and gender

There were significant differences in BMI SD, fasting glucoses, triglycerides, and hemoglobin A1c among ethnic groups. While African American subjects had higher BMI SDS and hemoglobin A1C values compared to Caucasians, fasting serum glucose levels were found to be significantly higher among Caucasians compared to African-American patients (Table 3).

**Table 3:** Clinical and biochemical Characteristics of participants according to ethnicity/race

Ethnicity	Caucasians	African American	P
N	28	72	
Age	13.9 ± 2.0	13.9 ± 2.1	NS
Gender (% Female)	57%	57%	NS
Hypertension %	19%	17%	NS
BMI SDS	2.2 ± 0.4	2.4 ± 0.3	0.034
Waist Circumference (cm)	104.3 ± 17.6	109.6 ± 14.3	NS
Fat Mass (kg)	37.8 ± 14.3	44.7 ± 18.3	NS
Glucose (mmol/L)	4.9 ± 0.5	4.7 ± 0.5	0.01
Insulin (pmol/L)	205.6 ± 162.0	193.0 ± 157.9	NS
HOMA-IR	6.5 ± 5.7	5.6 ± 4.6	NS
hs-CRP (mg/L)	2.75 ± 2.09	2.76 ± 2.19	NS
SUA (mg/dL)	336.9 ± 59.7	339.7 ± 87.0	NS
Cholesterol (mmol/L)	4.0 ± 1.1	4.0 ± 0.7	NS
Triglycerides (mmol/L)	1.43 ± 0.71	1.08 ± 0.49	0.006
Triglycerides:HDL-C ratio	2.95 ± 1.9	2.29 ± 1.3	NS
Prevalence of MS (%)	39%	26%	NS

Abbreviations: fat mass (FM), homeostatic model assessment estimates for insulin resistance (HOMA-IR), metabolic syndrome (MS), not applicable (NA) and not significant (NS)

Fourty-3 % of the cohort were male and 57% were female (Table 4). Female subjects had higher pubertal maturation staging of the males and females. Female subjects were found to have significantly higher fat percentage, lower FFM/FM ratio (data not shown) whereas males were found to have higher average fasting serum glucose levels and AST/ALT ratio (data not shown).

**Table 4:** Clinical and Biochemical Characteristics of Participants Based on Gender Abbreviations: fat mass (FM), homeostatic model assessment estimates for insulin resistance (HOMA-IR), metabolic syndrome (MS), not applicable (NA) and not significant (NS)

Parameters	All	Male	Female	*P*
N	100	43	57	NA
Age (yrs)	13.9 ± 2.0	13.6 ± 1.9	14.2 ± 2.1	NS
Tanner Stage	4.0 ± 1.3	3.4 ± 1.6	4.3 ± 0.9	0.005
Race/Ethnicity				
Caucasians (%)	28%	28%	28%	NS
African Americans (%)	72%	72%	72%	NS
Hypertension %	17%	14%	19%	NS
BMI SDS	2.4 ± 0.4	2.4 ± 0.3	2.3 ± 0.4	NS
Waist circumference	104.5 ± 18.0	108.6 ± 14.5	107.8 ± 16.1	NS
Fat Mass (kg)	42.8 ± 17.6	38.1 ± 14.3	46.3 ± 18.9	0.015
Glucose (mmol/L)	4.8 ± 0.5	5.0 ± 0.4	4.6 ± 0.5	0.001
HbA_1c_ (%)	5.5 ± 0.3	5.5 ± 0.3	5.5 ± 0.2	NS
Insulin (pmol/L)	194.4 ± 157.2	187.7 ± 145.6	199.5 ± 166.6	NS
HOMA-IR (mol uU/mL)	5.8 ± 4.9	5.9 ± 4.9	5.8 ± 4.9	NS
AST	29.7 ± 10.5	31.2 ± 9.8	28.6 ± 11.0	NS
ALT	35.8 ± 16.1	38.7 ± 16.0	33.6 ± 16.0	NS
hs-CRP (mg/L)	2.8 ± 2.2	2.8 ± 2.3	2.8 ± 2.0	NS
SUA (umol/L)	341.5 ± 76.3	357.7 ± 75.8	329.3 ± 75.0	NS
Cholesterol (mmol/L)	4.0 ± 0.8	3.9 ± 0.7	4.2 ± 0.9	NS
Triglycerides (mmol/L)	1.2 ± 0.6	1.1 ± 0.53	1.2 ± 0.62	NS
Triglycerides:HDL-C ratio	2.5 ± 1.5	2.5 ± 1.4	2.5 ± 1.6	NS
Prevalence of MS (%)	30%	23%	35%	NS

### Findings in the entire cohort

**Fig. 1:**
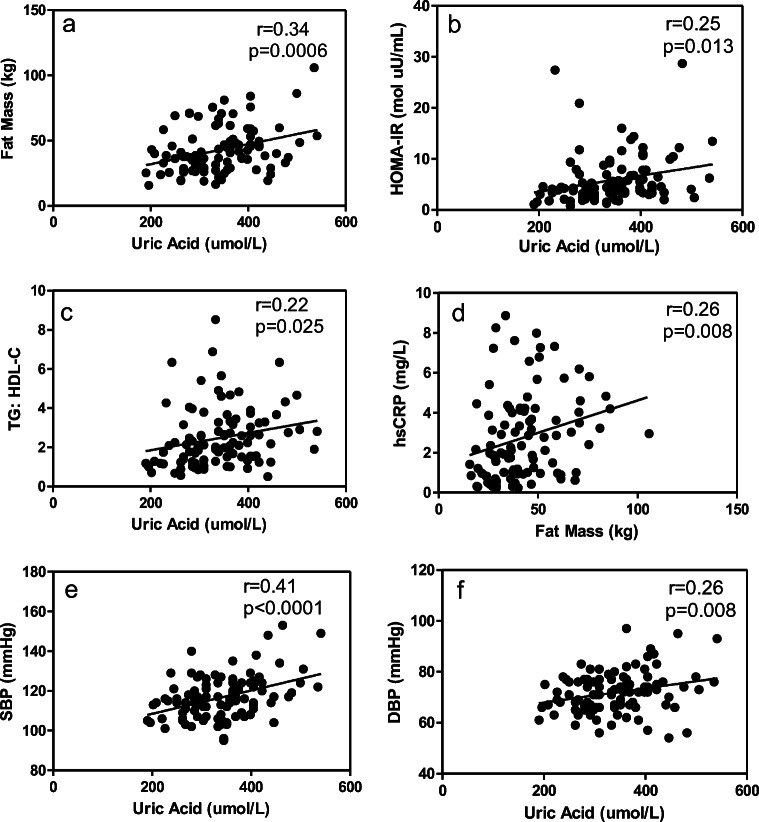
Spearman correlations between uric acid and markers of metabolic syndrome. A. uric acid vs. fat mass. B. uric acid vs. HOMA-IR C. uric acid vs. triglyceride:HDL ratio D. High-sensitivity CRP vs. Fat mass. E. uric acid vs. systolic blood pressure F. uric acid vs. diastolic blood pressure

Table 5 provides a summary of bivariate correlations among the clinical and biochemical variables for the entire cohort. FM was found to correlate with SBP and DBP as well as HOMA-IR, triglycerides:HDL-C ratio, and hsCRP. SUA were shown to correlate with FM, HOMA-IR, triglycerides:HDL-C levels, systolic and diastolic blood pressure (Fig. 1). hsCRP was not significantly positively correlated with SUA in the entire cohort. Interestingly, hs-CRP was not correlated with the triglcyerides:HDL or HOMA-IR. All patients had a GFR >60 ml/min/1.73 m^2^. Mean GFR was 107 ml/min/1.73 m^2^.

**Table 5:** Bivariate correlations of clinical and biochemical characteristics for whole cohort of participants

	FM (kg)	SBP (mmHg)	DBP (mmHg)	SUA (umol/L)	TG: HDL-C	HbA_1c_ (%)	HOMA-IR (mol uU/mL)
FM (kg)							
SBP (mmHg)	0.192						
DBP (mmHg)	0.323***	0.616***					
SUA (umol/L)	0.335***	0.414**	0.263**				
TG: HDL-C	0.208*	.225*	0.076	0.224*			
HbA_1c_ (%)	0.049	0.099	0.076	0.149	−0.011		
HOMA-IR (mol uU/mL)	0.237*	0.319***	0.202	0.247*	0.228*	0.196	
hs-CRP (mg/mL)	0.263**	0.055	0.034	0.06	0.05	−0.114	−0.046

*p < 0.05; **p < 0.01; *** *p* < 0.001

Six subjects were identified with impaired fasting glucose. These patients had significant elevation in HOMA-IR, fasting serum glucose and serum A1c compared to those without impaired glucose tolerance. Interestingly, looking at these patients, uric acid positively correlated with insulin levels, FM, HOMA-IR, SBP and DBP. High-sensitivity CRP correlated with FM but was not positively correlated to SUA (Fig. 1).

## Discussion

In the present study, elevated SUA was identified in 46% of the entire cohort and was positively correlated with higher triglcyerides;HDL-C and HOMA-IR, an index of insulin resistance. Females were found to have significantly higher FM and serum insulin levels than males. Patients with MS were shown to have significantly higher SUA as well as significantly higher SBP and DBP, fasting serum insulin and cholesterol. Patients with hyperuricemia had a higher prevalence of MS. These findings are consistent with prior studies showing that hyperuricemia is an independent risk factor associated with the development of MS [18, 30].

Hyperuricemia can impair vascular function by exerting pro-oxidant effects and by decreasing NO bioavailability which likely induces hypertension and MS [31]. In adults, SUA has been seen as a biomarker for cardiometabolic risk [32]. Prior studies in adults have shown that participants with elevated SUA had a higher incidence of diabetes and pre diabetes in adults [33]. Studies has shown that hyperuricemia alone, independent of obesity, can increase the risk of hypertension [34]. Indeed, SUA was found to be associated with elevated blood pressure in our cohort. Studies which have looked at the incidence of gout determined that obesity was independently associated with gout and hyperuricemia in women [35, 36] Our current study also reinforces that those with MS had increased SUA levels.

In our study, the incidence of hyperuricemia was observed in 39% in our population. The prevalence of hyperuricemia among adolescents with MS is variable among different populations [37]. Ford et al. reported hyperuricemia prevalence of 59.9% among US children and adolescents using SUA level of 334 uM. Tang et al. observed a prevalence of 30.5% in Japanese children using a SUA cutoff level of 416 uM (7.0 mg/dL) for male and 369 (6.2 mg/dL) uM in males and females. These studies show incidences which are different from in our study, most likely due to different SUA cutoffs. In our study, those with MS, SUA levels were significantly higher compared to those patients who did not meet criteria for MS. These findings are consistent with a prior study that looked at adolescents and hyperuricemia [38]. Similar to other studies, no gender differences in the prevalence of hyperuricemia in study participants were observed. Limited data in adolescents is available for evaluation of the relationship between hyperuricemia and the connection with inflammation in MS.

Of note, there were differences in the ethnicities between glucoses and hemoglobin A1C in our study with white participants having higher fasting glucose levels with African-American participants having a higher hemoglobin A1C values. Based on other studies, African-American persons have higher hemoglobin A1C levels across the full spectrum of glucose levels secondary to differences in rates of glucose transport and glycosylation of the erythrocyte membrane [39, 40]. This difference could explain the findings in our study regarding average hemoglobin A1C.

Prior studies have shown that there is an increased incidence of elevated hsCRP in those who are obese. Our study did not show direct correlation between SUA and hsCRP, as other studies had shown. Our postulation is that perhaps the assay for hsCRP used during our study was different from the assay used in other studies. Another potential explanation is that with our study, since we were only evaluating obese patients, there was not enough variation in the SUA to detect a correlation of hsCRP because the values were all at one end of the spectrum.

Limitations to this study include retrospective design of the study and lack of glucose tolerance data to assess glucose homeostasis and beta-cell function and its relationship with SUA. In our study, there were also no age and sex-matched normal weight controls for each racial group to assess the correlation of hsCRP to SUA. These controls may help assess correlations between inflammatory markers and hyperuricemia. In addition, the accuracy of bioelectrical impedance analysis (BIA) for assessment of body composition has been questioned because of larger errors in individual estimates of body composition in large groups of normal weight or obese pediatric subjects compared with DXA [41]. However, BIA has been deemed accurate and has been previously shown to correlate DXA to measure body fat percentage in normal and overweight patients. [42–44] However, recent studies have suggested BIA can under-estimate body fat in obese Asian individuals, which could be a limitation of this method. [45] Since we did not have any subject of Asian background, this limitation is not applicable. Therefore, we utilized the body impedance scale to assess body fat percentage. Exclusion of conditions which may affect uric acid was not done for this study, therefore it is a limitation and further study may be needed to look specifically into conditions such as asthma.

In conclusion, patients with hyperuricemia demonstrated significant elevations in markers of metabolic syndrome including higher blood pressures, serum glucoses, insulin resistance and triglycerides. Our study showed that patients with hyperuricemia have significantly higher fat mass on average. Our study confirmed the monotonic correlation of fat mass and uric acid in our population, which was composed predominately of African-American patients, which may not reflect a linear correlation. Additional studies are needed to further understand the relationship between hyperuricemia and markers of inflammation in the adolescent population with and without metabolic syndrome.
